# Molecular Characterization and SNP Detection of CD14 Gene of Crossbred Cattle

**DOI:** 10.4061/2011/507346

**Published:** 2011-10-25

**Authors:** Aruna Pal, Arjava Sharma, T. K. Bhattacharya, P. N. Chatterjee, A. K. Chakravarty

**Affiliations:** ^1^Animal Genetics Division, Indian Veterinary Research Institute, Izatnagar, Pin-243122, India; ^2^Project Directorate on Poultry, Rajendranagar, Hyderabad, India; ^3^Animal Nutrition Division, Indian Veterinary Research Institute, Izatnagar, Pin-243122, India; ^4^Dairy Cattle Breeding Division, National Dairy Research Institute, Karnal, Haryana, Pin-132001, India

## Abstract

CD14 is an important molecule for innate immunity that can act against a wide range of pathogens. The present paper has characterized CD14 gene of crossbred (CB) cattle (*Bos indicus*×*Bos taurus*). Cloning and sequence analysis of CD14 cDNA revealed 1119 nucleotide long open reading frame encoding 373 amino acids protein and 20 amino acids signal peptide. CB cattle CD14 gene exhibited a high percentage of nucleotide identity (59.3–98.1%) with the corresponding mammalian homologs. Cattle and buffalo appear to have diverged from a common ancestor in phylogenetic analysis. 25 SNPs with 17 amino acid changes were newly reported and the site for *mutational hot-spot* was detected in CB cattle CD14 gene. Non-synonymous substitutions exceeding synonymous substitutions indicate the evolution of this protein through positive selection among domestic animals. Predicted protein structures obtained from deduced amino acid sequence indicated CB cattle CD14 molecule to be a receptor with horse shoe-shaped structure. The sites for LPS binding, LPS signalling, leucine-rich repeats, putative N-linked glycosylation, O-linked glycosylation, glycosyl phosphatidyl inositol anchor, disulphide bridges, alpha helix, beta strand, leucine rich nuclear export signal, leucine zipper and domain linker were predicted. Most of leucine and cysteine residues remain conserved across the species.

## 1. Introduction

Manipulation of the host immune response is the most precise and effective tool to lower down the disease incidences and to nullify the limitations associated with antibiotic treatment or vaccination. CD14 is an important molecule for innate immunity. CD molecule ranges from 1 to 166 with differential structure and functions [1], of these CD14 is the most important molecule known so far, playing a vital role against several enterotoxigenic bacteria. Its pattern recognition receptor binds mainly with LPS (lipopolysaccharide), lipotechoic acid, arachidonic acid and thus releases various cytokines which act for body's defence. Body's immunity thus can act against a wide range of pathogens including gram-negative bacteria and gram positive as *Mycobacterium *sp., *Pseudomonas *sp. and *Staphylococcus aureus*. Soluble CD14-enriched bovine colostrums and milk induces B cell growth and differentiation [2]. CD14 functions both as a cell membrane receptor and a soluble receptor for bacterial LPS. It has been considered as an important molecule for its role on various diseases, like mastitis [3], treponemiasis [4] and glomerulonephritis [5]. 

CD14 gene may be manipulated in various ways for disease management. Recombinant protein may be used as therapeutic agent; cloned gene insertion may be utilized for somatic gene therapy and by the development of transgenic disease resistant animals. Detection of SNP is useful for analysis of the evolutionary history of species development, assessment of biodiversity, associative studies between polymorphisms, and disease-resistance. Scanty reports are available so far for genetic polymorphism of CD14 gene in an animal at coding region; however, CD14 gene polymorphism study at promoter region is available in human [6–8]. 

In recent days, crossbred (CB) cattle are gaining much importance being high milk yielder compared to indigenous cattle in developing countries. CB cattle arise due to crossing of low yielding *Bos indicus* with high yielding *Bos taurus*, preferably Holstein Friesian or Jersey. In India, CB cattle constitute 13.3% of the total cattle population with high growth rate for CB milch cattle (34.4%), whereas indigenous milch cattle has decreased by 6.1% [9]. There is a tremendous increase in the CB cattle in the country that is, 22.8% but the indigenous cattle declined by 10.2% [9]. However, the major drawback was that CB cattle are poor in adaptability and disease resistance compared to indigenous cattle. So there is an immediate need to improve disease resistance trait in CB cattle. Scanty reports are available regarding CD14 gene in *Bos taurus* [10, 11]. So far no reports are available regarding molecular characterization of CD14 gene in CB cattle or its protein structure.

Keeping the above facts in mind, the present investigation has been planned to clone and sequence the CD14 gene of crossbred cattle, study of CD14-derived peptide using bioinformatics tools, to compare the sequence homology with other species, and to identify the SNP of CD14 gene of CB cattle.

## 2. Materials and Methods

### 2.1. Animals, Sample Collection, and RNA Isolation

Blood was collected aseptically by jugular vein puncture over 2.7% ethylenediamine tetraacetic acid from healthy CB cattle. Peripheral blood mononuclear cells (PBM cells) were isolated from whole blood by density gradient centrifugation using Lymphocyte Separation Media (LSM) (Himedia) [12]. The total RNA was isolated from separated cells using TRIzol (Life Technologies, USA), following manufacturer's instructions and was further used for cDNA synthesis.

### 2.2. Synthesis and Confirmation of cDNA PCR Amplification of CD14 Gene

The 20 *μ*L reaction mixture contained 5 *μ*g of total RNA, 0.5 *μ*g of oligo dT primer (16–18 mer), 40 U of Ribonuclease inhibitor, 1000 M of dNTP mix, 10 mM of DTT, and 5 U of MuMLV reverse transcriptase in reverse transcriptase buffer. The reaction mixture was gently mixed and incubated at 37°C for 1 hour. The reaction was stopped by heating the mixture at 70°C for 10 minutes and chilled on ice. The integrity of the cDNA checked by PCR. To amplify full length open reading frame (ORF) of CD14 gene sequence, a specific primers pair was designed based on the CD14 mRNA sequences of cattle (Accession No-Acc No. AF141313) by DNASIS MAX software (Hitachi Miraibio Inc., USA). The primers were Forward: CD14-1-F ATGGTCTGCGTGCCCTACCTG and Reverse: CD14-70-1-R GGAGCCCGAGGCTTCGCGTAA. 25 *μ*L reaction mixture contained 80–100 ng cDNA, 3.0 *μ*L 10X PCR assay buffer, 0.5 *μ*L of 10 mM dNTP, 1 U Taq DNA polymerase, 60 ng of each primer, and 2 mM MgCl_2_. PCR-reactions were carried out in a thermocycler (PTC-200, MJ Research, USA) with cycling conditions as, initial denaturation at 94°C for 3 min, denaturation at 94°C for 30 sec, annealing at 61°C for 35 sec, and extension at 72°C for 3 min were carried out for 35 cycles followed by final extension at 72°C for 10 min.

### 2.3. cDNA Cloning and Sequencing

PCR amplicons verified by 1% agarose gel electrophoresis were purified from gel using Gel extraction kit (Qiagen GmbH, Hilden, Germany) and ligated into pGEM-T easy cloning vector (Promega, Madison, WI, USA) following manufacturers' instructions. The 10 *μ*L of ligated product was directly added to 200 *μ*L competent cells, and heat shock was given at 42°C for 45 sec. in a water bath, and cells were then immediately transferred on chilled ice for 5 min., and SOC was added. The bacterial culture was pelleted and plated on LB agar plate containing Ampicillin (100 mg/mL) added to agar plate @ 1 : 1000, IPTG (200 mg/mL) and X-Gal (20 mg/mL) for blue-white screening. Plasmid isolation from overnight-grown culture was done by small-scale alkaline lysis method described by Sambrook and Russell [13]. Recombinant plasmids were characterized by PCR using gene-specific primers and restriction enzyme digestion based on reported nucleotide sequence for cattle. The enzyme EcoR I (MBI Fermentas, USA) isused for fragment release. CD14 gene fragment insert in recombinant plasmid was sequenced by automated sequencer (ABI prism) using dideoxy chain termination method with T7 and SP6 primers (Chromous Biotech, Bangalore).

### 2.4. Sequence Analysis

The nucleotide sequence so obtained was analyzed for protein translation, sequence alignments, and contigs comparisons by DNASTAR Version 4.0, Inc., USA. Novel sequence was submitted to the NCBI Genbank and accession number (GU368102) was obtained which is available in public domain now.

### 2.5. Phylogenetic Analysis

Phylogenetic analysis was also performed to determine the evolutionary relationship of CB cattle with other species. Nucleotides sequences were then aligned with that of the reported CD14 sequences of different species including *Bubalus bubalis* (DQ457089), *Bos taurus (*NM_174008), *Canis familiaris* (XP_848746), *Capra hircus* (DQ457090), *Homo sapiens* (NM_000591), monkey (XP_517975), *Mus musculus* (NM_009841), *Rattus norvegicus* (NP 068512), *Gallus gallus* (AM933591), *Equus caballus* (AF200416), *Macaca mulatta *(NM_001130433) *Sus scrofa* (EF051626), *Ovis aries *(AY289201), and *Oryctolagus cuniculus *(M85233.1) using the ClusterW method of multiple alignment which is slow and accurate (MegAlign Programme of Lasergene Software, DNASTAR).

### 2.6. Study of Predicted CB Cattle CD14 Protein Using Bioinformatics Tools

Predicted peptide sequence of CD14  gene of CB cattle was derived by Edit sequence (Lasergene Software, DNASTAR) and then aligned with the CD14 peptide of other livestock and avian using Megalign sequence Programme of Lasergene Software (DNASTAR). Prediction of signal peptide of CD14 gene was conducted using the software (Signal P 3.0 Sewer-prediction results, Technical University of Denmark). Leucine percentage was calculated manually from predicted peptide sequence. Di-sulphide bonds were predicted using suitable software (http://bioinformatics.bc.edu/clotelab/DiANNA/) and by homology search with other species CD14 polypeptide [14].

Protein sequence level analysis study was carried out with specific software (http://www.expasy.org./tools/blast/) for determination of leucine rich repeats (LRR), leucine zipper, N-linked glycosylation sites, detection of Leucine-rich nuclear export signals (NES), and detection of the position of GPI anchor. Detection of Leucine-rich nuclear export signals (NES) was carried out with NetNES 1.1 Server, Technical university of Denmark. Analysis of O-linked glycosylation sites was carried out using NetOGlyc 3.1 server (http://www.expassy.org/), whereas N-linked glycosylation site were detected by NetNGlyc 1.0 software (http://www.expassy.org/). Regions for alpha helix and beta sheet were predicted using NetSurfP-Protein Surface Accessibility and Secondary Structure Predictions, Technical University of Denmark [15]. Domain linker prediction was done according to the software developed [16]. LPS-binding site [17] as well as LPS-signaling sites [18] were predicted based on homology studies with other species CD14 polypeptide. 3D model of CD14 polypeptide was predicted based on Swissmodel repository [19]. 

## 3. Results and Discussion

### 3.1. Cloning and Characterization of CD14 cDNA

**Figure 1:**
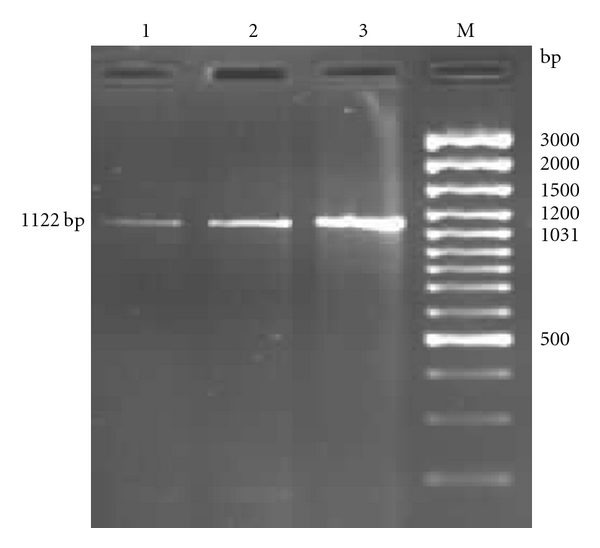
Amplification of 1122 bp fragment of CD14 gene of crossbred cattle by RT-PCR. Lanes 1 and 2: Amplified product from cDNA of CD14 gene. Lane 3: Confirmation of insert by plasmid PCR. Lane M: 100 bp DNA ladder plus.

**Figure 2:**
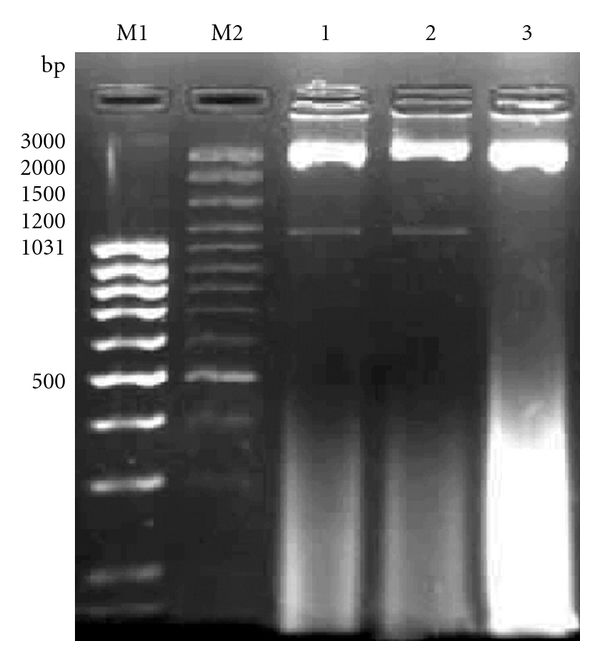
Release of 1122 bp insert of CD14 gene by EcoRI digestion. Lane M1: 100 bp DNA ladder. Lane M2: 100 bp DNA ladder plus. Lane 1: Release of 1122 bp insert of CD14 gene by plasmid PCR. Lane 2: Release of 1122 bp insert of CD14 gene by plasmid after *RE *digestion. Lane 3: Control representing Original plasmid without recombinant CD14 insert.

CD14 gene of CB cattle was observed to be 1.12 kb when checked in 1% agarose gel electrophoresis (Figure 1). The fragment was cloned into pGEM-T Easy Vector. For the confirmation of insert, the transformants were screened using colony PCR followed by plasmid PCR and RE digestion of plasmid with *EcoR-I* as restriction enzyme released the insert of approximately 1.12 kb from the plasmid (Figure 2). 

CD14 gene of CB cattle amplified was observed to be 1122 bp. Since this is the first report for study of CD14 gene in CB cattle, comparison was not possible. However, similar nucleotide length was observed in *Bos taurus* [11, 20, 21], *Capra hircus* [22], and *Bubalus bubalis* [23]. 

**Figure 3:**
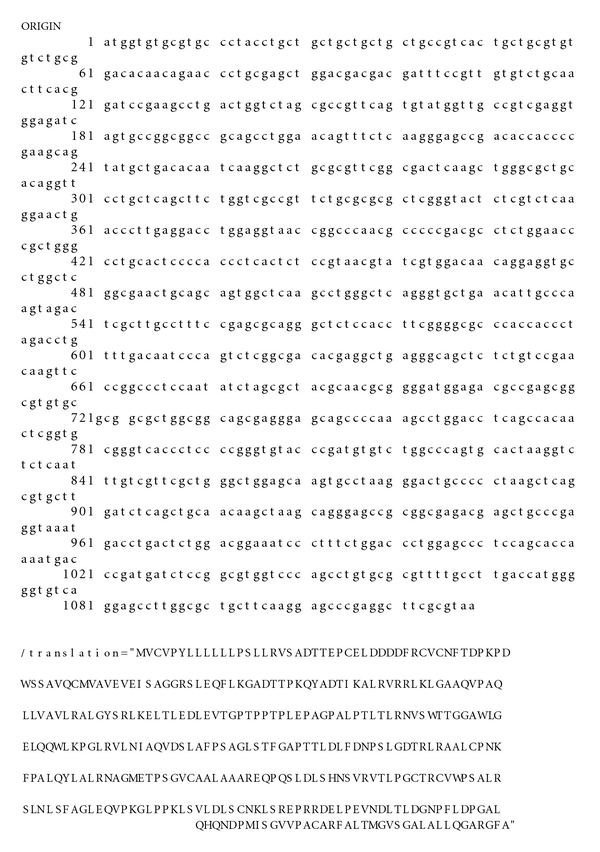
Nucleotide and amino acid sequence of CD14 gene of cattle (GU368102).

The DNA insert of CD14 gene was found to be exactly 1122 bp with ATG as start codon followed by an open reading frame of 1119 nucleotides, after sequencing of a selected representative clone (Figure 3). The comparison of obtained nucleotide sequence and derived amino acid sequence using multiple alignment (Cluster W, slow and accurate) with available CD14 nucleotide sequence of cattle and buffalo confirmed that the insert was CD14 gene. GC content of CB cattle CD14 gene was found to be as high as 62.57%, similar to *Bos taurus *(62.75%). This is slightly more than *Bubalus bubalis* (62.3%) and *Capra hircus* (62.2%), slightly less than *Homo sapiens* (62.94%). GC content of CB cattle is much higher than *Rattus norvegicus *(56.12%) and lower than *Canis familiaris* (64.07%).

### 3.2. Phylogenetic Analysis of CD14 Gene of CB Cattle with Other Species

**Figure 4:**
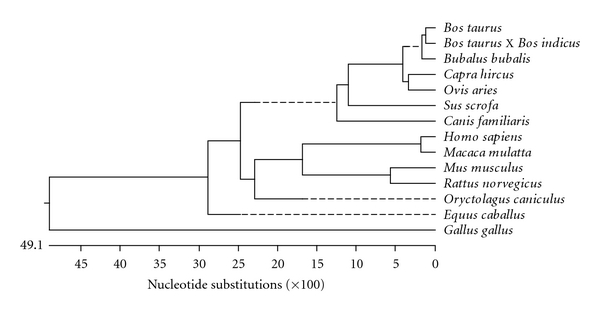
Phylogenetic analysis of crossbred cattle (*Bos indicus X Bos taurus*) with other species.

CB cattle CD14 gene is 98.1, 96.0, 94.7, 90.5, 81.5, 81.1, 78.2, 78.1, 76.5, 70.6, 68.5, 59.3, 11.0% identical to *Bos taurus, Bubalus bubalis, Ovis aries, Capra hircus, Sus scrofa, Equus caballus, Homo sapiens, Canis familiaris, Oryctolagus cuniculus, Mus musculus, Rattus norvegicus, Macaca mulatta, *and *Gallus gallus CD*14 gene (Table 1). Phylogenetic analysis of CB cattle with *Bos taurus* and other species indicates that CB cattle and *Bos taurus* are genetically the most similar followed by *Bubalus bubalis* (Figure 4). Phylogenetic closeness of buffalo with cattle as observed in the present study has also been reported while studying other genes like growth hormone gene [24]. *Bos taurus *CD14 gene sequence when compared to other species, also revealed that cattle was phylogenetically close to buffalo [20]. Comparative sequencing of nucleotide sequences of crossbred cattle (*Bos indicus X Bos taurus) *with * Bubalus bubalis *revealed similar results [23, 25]. Phylogenetic analysis of CB cattle with *Bos taurus* and other species indicates that CB cattle and *Bos taurus* are genetically most similar (98.1%), and thus grouped together. 1.9% dissimilarity between CB cattle and *Bos taurus* may be due to the inheritance of *Bos indicus* in CB cattle.

In other words, ruminants were found to be phylogenetically close and genetically similar. CB cattle were found to be the most distant to mouse and rat among the mammalian species studied. Thus it is evident that CB cattle has evolved from an ancestor which is genetically much distant than that of *Rattus norvegicus* or *Mus musculus*. *Canis familiaris* and *Homo sapiens* were also genetically distant from CB cattle. *Gallus gallus* has been found to be genetically most distant to CB cattle.

### 3.3. Study of Predicted CB Cattle CD14 Protein Using Bioinformatics Tools

The predicted peptide sequence of CD14 gene of crossbred cattle revealed 373 amino acids precursor corresponding to coding sequence of CD14 gene (Figure 3) and a 20 amino acid signal peptide. This is similar to *Bos taurus* [11] and *Capra hircus* [22], where CD14 peptide also contains 373 amino acids. CB cattle CD14 peptide is of higher Mol wt. (39939.29 Daltons) than *Bos taurus* (39679.96 Daltons) and *Bubalus bubalis* (39705.07 Daltons). CB cattle CD14 peptide sequence was characterized by the presence of two extra strongly basic amino acids and one extra strongly acidic amino acid than *Bos taurus*, whereas less by one hydrophobic and one polar amino acid than *Bos taurus.*

**Figure 5:**
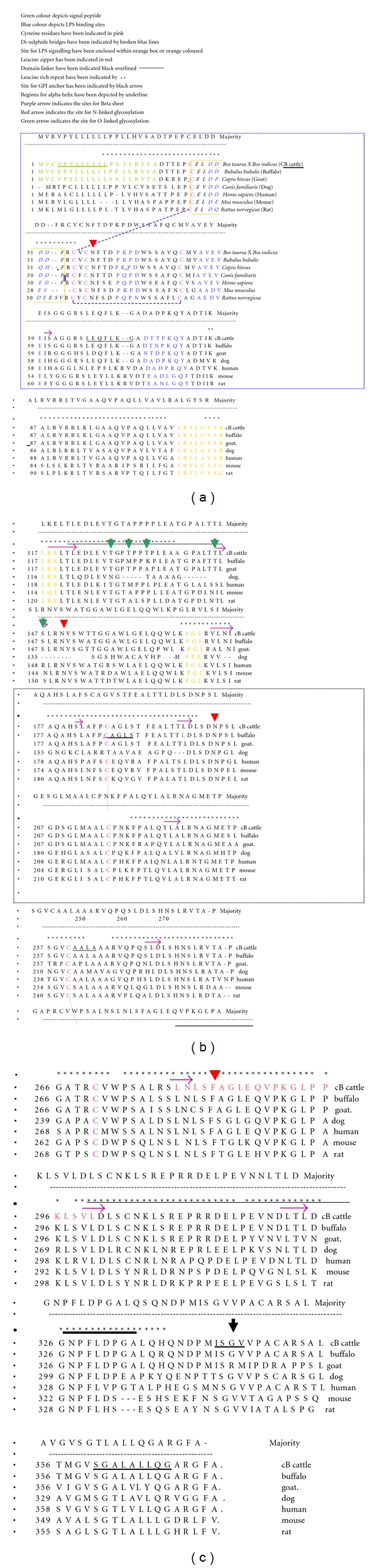
Alignment of CB cattle CD14 derived peptide with other species.

Protein sequence level analysis study revealed that nine leucine rich repeats (LRR) have been identified in predicted peptide sequence of CB cattle CD14 cDNA (Table 2, Figure 5). These LRRs are considered to participate in receptor and ligand interactions and have various cellular functions during early phases of the immune response [26]. Comparison of CB cattle CD14 gene with other spp. revealed that *Homo sapiens*, *Mus musculus*, *Bos taurus*, *Capra hircus,* and *Bubalus bubalis* was reported to have 10, 10, 10, 7, and 6 LRR, respectively [23, 27]. It has already been reported that LRR in extracellular domain is responsible for the recognition of pathogens. Crystal structure of *Mus musculus* CD14 gene has been depicted with the grooves responsible for receptor recognition and binding [14]. Since CD14 molecules can bind to a wide range of substances including lipopolysaccharides from gram-negative bacteria, lipoarabinomannan of mycobacteria, mannuronic acid polymers of *Pseudomonas sp.,* and to peptidoglycans of *Staphylococcus aureus*, it is expected that there should be sufficient variability in the pathogen recognition and receptor binding site, which is primarily comprised of LRR region of the CD14 molecule. Thus, the presence of LRR coding region may be the region for maximum variability to enable the CD14 molecule to bind with a wide range of substances. From the sequence alignment studies for the derived amino acid sequences for different species, it was observed that the particular amino acid leucine was almost unaltered within the leucine rich repeats, and the variations were observed for other amino acids. Although there are no reports regarding the number of LRR and its relation to disease susceptibility, a trend has been observed with more the species is resistant, less the no. of LRR. Buffaloes were reported to have least number of LRR among the species under study. As reported from a separate study, buffalo is most resistant [28] among common farm animals, namely, cattle, goat, sheep, and buffalo. So, it may be possible that the lesser number of LRR, the more it is resistant to diseases. However, further studies the species possess are needed for its confirmation.

The CD14 derived-peptide sequence of crossbred cattle contained 4 putative N-linked glycosylation sites (Figure 5, Table 3) whereas, there is report of three, five, four, five, and four glycosylation sites for N-linked glycosylation in *Bos Taurus *[29], *Mus musculus* [30], *Homo sapiens* [30], *Rattus norvegicus* [31], *Bubalus bubalis* [23], respectively. N-linked glycosylation is vital for the molecule as it supports the molecule to be present either in membranous or soluble form. 

CB cattle contain 5 sites for O-linked glycosylation (Figure 5) at amino acid positions 128, 131, 134, 145, 147, which differs from *Bubalus bubalis* containing 3 sites [23]. However, O-linked glycosylation was reported to be absent in *Homo sapiens* purified native CD14 molecule [32]. Glycosylation is needed when hydrophilic clusters of carbohydrates alter the polarity and solubility of protein or protein folding.

Protein sequence level analysis study revealed that in CB cattle, there was a glycosyl phosphatidyl inositol (GPI) anchor located at C-terminus near 345th position of the CD14 molecule (Figure 5). It differs from *Bubalus bubalis* containing glycosyl phosphatidyl inositol (GPI) anchor located at C-terminus near 353rd position of the CD14 molecule [23]. Similar findings were reported by Wright [33] in other species like and *Mus musculus.* GPI anchor is vital for the molecule as the basic function of GPI in mammalian cell system is to make a bridge between CD14 and cell surface. However, in case of avains, CD14 is trans-membrane rather than GPI anchored [34]. 

Alignment of derived peptide sequence of CB cattle with other species (Figure 5) revealed that leucine residues are conserved across the species (Figure 5). Cystine residue has also been observed to be conserved across species (Figure 5). Similar observations were also observed for *Mus musculus* [14].

 Five disulphide bridges have been predicted between cysteine residues in CD14 polypeptide of CB cattle of which only four were visualized in Figure 5. Since Cys287 is a tyrosine in the mouse CD14 sequence, in the homology study of CD14 sequences for different species (Figure 5), the fifth disulphide bond is not visualized. Disulphide bridges are responsible for protein folding. Protein folding in turn gives rise to groove formation for binding site for ligand. *Homo sapiens* CD14 also contains 5 disulphide bonds [32]. *Mus musculus* CD14 molecules were reported with 4 disulphide bonds [14]. The crystal structure of mouse CD14 also sheds light on the sites of cotranslational modifications of human CD14. Similar five disulfide bonds are implicated from the biochemical studies in human [35]. In human CD14, a differential impact is observed for the five disulfide bonds on CD14 folding, with the first two being indispensable, the third and fourth being important, and the last (Cys287–Cys333) being dispensable. When mapped to the crystal structure of mouse CD14, the first two disulfide bonds are found in the *β* sheets in the inner concave surface (the “core” structure), whereas the third and fourth disulfide bonds are in the loops and helices on the outer surface (the “peripheral” structure). The last disulfide bond (Cys287–Cys333) is not seen in the structure, because the construct used for crystallization has the truncation of C-terminal 33 amino acids, and Cys287 is a tyrosine in the mouse CD14 sequence [14]. The first disulfide bond is therefore essential for the structural and functional integrity of CD14. The third and fourth disulfide bonds contribute to but do not determine CD14 folding, because the substitutions of these cysteines with alanines decrease but do not abrogate CD14 secretion. The last disulfide bond (Cys287–Cys333) has no effect on CD14 folding [32].

In CB cattle, four LPS-binding sites were depicted from 29th–32nd, 44th–47th, 55th–59th, and 75th–80th amino acid positions (Figure 5). LPS-binding site region I overlaps with LRR. The structural characteristics of the binding site may explain the broad ligand specificity of CD14. Although the hydrophobic bottom and walls of the pocket are rigid, the generous size of the pocket may allow structural variation in the hydrophobic portion of the ligand. Structural diversity in the hydrophilic part of the ligands could be explained by the considerable flexibility of the hydrophilic rim combined with the multiplicity of grooves available for ligand binding. This is also the case of *Mus musculus* CD14 molecule reported by Sambrook and Russell [13]. LPS-binding site is in agreement with the present study that N-terminal region of Homo* sapiens* CD14 molecule is responsible for LPS binding [36]. 

Three LPS signaling sites were predicted for amino acid positions as 27th–33th, 109th–119th, and 169th–171th amino acid (Figure 5). Similar observations were reported by Sambrook and Russell [13] in *Mus musculus *with four LPS-binding sites and three LPS signaling sites. Region I for LPS signaling site overlaps with LPS binding site. LPS signaling site also overlaps with LRR. This is similar to *Mus musculus* CD14 molecule, as reported by Sambrook and Russell [13].

In terms of secondary structure prediction, five regions were detected for *α* helix conformation as 4–12, 67–73, 241–244, 343–346, and 360–368, and eleven regions were identified for *β* strand conformation as 60-61, 118–122, 145–147, 173–176, 181-182, 197–199, 225–227, 252–254, 278–280, 299–301, 321–324 amino acid positions (Figure 5). However, in *Mus musculus*, 7 alpha helix, and 13 beta sheets were identified [13]. The monomeric subunit of CD14 contains eleven *β* strands, and 9 of them, from *β*2, *β*4 to *β*11, overlap with conserved leucine-rich repeats (LRRs) (Figure 5).

Leucine-rich nuclear export signals were detected for 7 sites at amino acid positions 12, 15, 16, 17, 117, 122, 127 (Figure 5). A nuclear export signal (NES) is a short *amino acid* sequence of 4 hydrophobic residues in a *protein* that targets it for export from the *cell nucleus* to the *cytoplasm* through the *nuclear pore* complex [37]. However, it is the first report of prediction of the site of leucine-rich nuclear export signal in CD14 gene in animal. 

DNA-binding motif as leucine zipper pattern was detected from amino acid position 279 of CB CD14 peptide (Figure 5), which is reported here for the first time in CD14 molecule. Leucine scissors [38] is a common three-dimensional structural motif in proteins. These motifs are usually found as part of a DNA-binding domain in various transcription factors and are therefore involved in regulating gene expression. The leucine zipper is a supersecondary structure that functions as a dimerization domain, and its presence generates adhesion forces in parallel alpha helices [39]. However, the site of leucine zipper in CD14 gene is predicted here for the first time in any animal. 

Domain linker site was predicted for amino acid positions 121–146 and 283–334 (Figure 5). Domain linker sequences are loop sequences connecting two structural domains [40]. linker are likely to act as a scaffold to prevent unfavourable interactions between folding domains [41]. Recent advances in protein engineering have come from creating multifunctional chimeric proteins containing modules from various proteins. These modules are typically joined via an oligopeptide linker, the correct design of which is crucial for the desired function of the chimeric protein. Analysis of the properties of naturally occurring interdomain linkers is useful with the aim to design linkers for domain fusion leading to chimeric protein formation [41]. 

CB cattle CD14 molecule is predicted to be mostly expressed on cell membrane or cell surface. Since CD14 is a receptor molecule, it is obvious to be expressed on cell surface. Moreover, in the present study, CD14 cDNA was prepared from mRNA expressed on cells of liver tissue. CD14 exists in two forms as membranous and soluble form [42, 43]. In the present study, CD14 cDNA cloned is the membranous form as it contains GPI anchor for the attachment with cell membrane as well as glycosylated form [42, 43]. 

**Figure 6:**
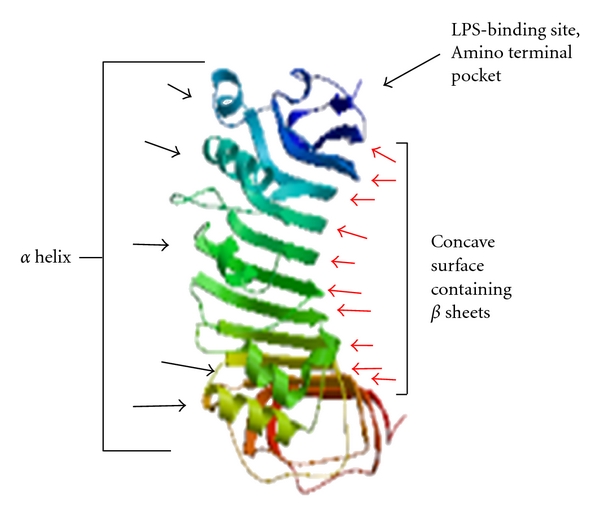
3D structure of CD14 peptide of crossbred cattle. It is a horse shoe shaped structure when exist as dimer. Since it is receptor molecule, presence of pockets and grooves for ligand binding. Blue end represents amino acid terminal with a pocket.Residue range: 24 to 331 amino acid of CD14 molecule. Model info: modelled residue range: 24 to 331 based on template *1wwlB* (2.50 Å) Sequence Identity (%): 62.581.

3D model of CD14 molecule of CB cattle was predicted from amino acid position 24 to 331 (Figure 6), with horseshoe-shaped structure, with alternating alpha helix and beta chains. CD14 exists as dimmer with the help of leucine zipper when acting as a receptor molecule. The finding is similar to study conducted by Sambrook and Russell [13], where they studied crystal structure of CD14 molecule of *Mus musculus*. The amino terminal pocket along with grooves is responsible for ligand binding. Since CD14 is a receptor, ligand binding is an important factor. Based on homology study for CD14 3D structure with *Mus musculus* [14] and *Homo sapiens *[32], LPS-binding pocket was predicted at the amino terminal end of CB cattle CD14 molecule, *α* helix were located at the convex surface, and *β* strand were located at the concave surface of horse-shoe shaped CD14 structure, represented in Figure 6.

### 3.4. SNP Study of CD14 Gene in Crossbred Cattle

CB cattle has resulted from the cross of *Bos taurus* and* Bos indicus. *As expected, 27 nucleotide changes were observed, when compared with the published sequences of CD14 gene of *Bos taurus*, out of which 25 were newly reported. 6 synonymous changes and 18 amino acid changes were reported out of which 17 were new changes (Table 4). Two SNPs for *Bos taurus* were reported previously [21]. Similar higher degrees of mutations have been observed in some other genes as 25 SNPs in Leptin gene in Korean cattle [44], 96 SNPs from TLRs, and their associated intracellular signaling molecules [45] in human.

Nonsynonymous substitutions exceeding synonymous substitutions indicate the evolution of this protein through positive selection among domestic animals. SNP for the CD14 gene of crossbred cattle was reported here for the first time.

An interesting observation is that the CD14 nucleotide sequence from 1 to 258 has no major SNP detected, except one A230C. This may be the reason that 1 to 60 nucleotide codes for signal peptide, when no variation is expected. The present finding is similar to *Bubalus bubalis*, when monomorphism was reported for this nucleotide region [20] except for one nucleotide change that is too within LPS-binding site. This variant containing threonine may be the species specific characteristics particular to CB cattle. This allele is most probably contributed by *Bos indicus*, since none of the *Bos taurus* contains this allele. 

Another interesting observation is that about 50% of the nucleotide changes, including 3 synonymous changes have been reported from 602nd to 800th nucleotide region of CD14 gene of CB cattle, that is, within 199 nucleotide. So, this region may be the hyper-variable region containing mutational hot spot. Similar findings were also reported in *Bubalus bubalis*, where 42 SNP were identified with 39 nonsynonymous changes, leading to 23 amino acid changes [20]. This region may be considered as the hypervariable region for buffalo also.

## 4. Conclusion

CD14 gene of crossbred cattle (*Bos indicus* crossed with *Bos taurus*) has been characterized for the first time in the present study. 25 SNPs with 17 amino acid changes were newly reported, and the site for *mutational hot-spot,* detected in CB cattle CD14 gene. CB cattle has been phylogenetic closest to *Bos taurus*. Cattle was observed to be phylogenetically closest to *Bubalus bubalis*. Hyper-variable regions containing *mutational hot spot* have been reported from 602nd to 800th nucleotide region of CD14 gene of CB cattle, that is, within 199 nucleotide. Further research need to be directed to find out the association of allelic variants with traits related to disease incidences, to establish it as marker. Gene insert containing the resistant variety of CD14 gene of CB cattle can be used for somatic gene therapy, particularly against mastitis. Transgenic animal production with CB cattle CD14 gene insert may provide the scope for future research to develop disease-resistant stock.

## Figures and Tables

**Table 1:** Percent identity of CD14 gene of crossbred cattle with other species.

∗∗∗	98.1	98.0	11.3	79.3	92.5	82.7	79.5	60.2	71.7	82.8	77.5	69.5	96.7	*Bos taurus*
2.3	∗∗∗	96.0	11.0	78.1	90.5	81.1	78.2	59.3	70.6	81.5	76.5	68.5	94.7	CB CATTLE
2.0	4.2	∗∗∗	19.0	78.7	92.0	82.3	79.5	60.1	71.7	82.6	77.2	69.4	96.3	*Bubalus bubalis*
83.5	86.5	82.7	∗∗∗	16.8	10.2	12.0	11.5	5.0	4.4	18.4	11.4	9.0	18.4	*Gallus gallus*
24.0	25.9	24.6	77.9	∗∗∗	75.7	81.6	77.8	53.1	68.0	79.5	75.5	68.0	80.3	* Canis familiaris *
7.9	10.3	8.5	83.2	29.2	∗∗∗	79.4	76.0	58.1	69.5	78.7	74.6	62.1	93.5	*Capra hircus*
20.7	23.3	21.1	79.8	20.8	25.2	∗∗∗	81.4	62.9	71.8	81.6	79.4	69.9	83.2	*Equus caballus*
24.5	26.6	24.2	79.2	26.3	29.4	22.0	∗∗∗	95.8	73.6	78.3	81.1	72.3	80.0	*Homo sapiens*
25.7	27.6	25.7	84.1	27.5	32.0	22.5	1.1	∗∗∗	57.0	59.6	62.6	57.0	60.5	MONKEY
36.6	39.0	36.7	97.2	39.2	41.0	34.5	33.1	33.5	∗∗∗	70.9	72.7	89.5	70.6	*Mus musculus*
19.9	22.2	20.1	75.8	24.6	25.9	22.7	26.9	28.4	38.6	∗∗∗	76.5	69.4	83.1	*Sus scrofa*
26.4	28.2	26.5	81.7	28.2	30.5	25.2	21.9	23.2	34.5	28.5	∗∗∗	70.1	78.2	*Oryctolagus cuniculus*
39.3	41.5	39.6	98.1	41.3	44.6	37.5	34.6	34.6	11.3	41.2	38.8	∗∗∗	69.7	*Rattus norvegicus*
3.4	5.7	3.8	81.0	22.7	7.0	20.1	24.0	25.7	36.6	19.6	25.0	39.2	∗∗∗	*Ovis aries*

Pair Distances of FINAL CB.meg ClustalW (Slow/Accurate, IUB); Percent Similarity in upper triangle; Percent Divergence in lower triangle.

**Table 2:** Leucine-rich repeats detected in derived peptide sequence of CD14 gene of crossbred cattle.

Repeat	From	To	Fragment
LRR	12	35	LPSLLRVSADTTEPCELDDDDFRC
LRR	86	107	KALRVRRLKLGAA.QVPAQLLVA
LRR	114	138	YSRLKELTLEDLEVTGPTPPTPLEP
LRR	176	200	IAQVDSLAFPSAGLSTFGAPTTLDL
LRR	220	242	FPALQYLALRNAGM.ETPSGVCAA
LRR	247	271	REQPQSLDLSHNSVRVTLPGCTRCV
LRR	273	296	PSALRSLNLSFAGLEQVPKGLPPK
LRR	298	316	SVLDLSCNKLSREPRRDEL
LRR	317	337	PEVNDLTLDGNPF..LDPGALQH

**Table 3:** N-linked glycosylation sites for derived CD14 peptide of crossbred cattle.

Position	Sequence
38	**N**FTD
150	**N**VSW
203	**N**PSL
280	**N**LSF

**Table 4:** SNP detection in CD14 gene of CB cattle and the amino acid changes.

	Position of SNP	Synm/Nonsynm	*Bos taurus*	*Bos taurus X Bos indicus*	Type of mutation	Resulting amino acid change
1	A230C	Non-Synm	A	C	Transversion	N77T
2	G363C	Synm	G	C	Transversion	
3	G412C	Non-synm	G	C	Transversion	A138P
4	G426A	Synm	G	A	Transition	
5	A430C	Non-synm	A	C	Transversion	T144P
6	G440C	Non-synm	G	C	Transversion	S147T
7	C536T	Non-synm	C	T	Transition	A179V
8	C538G	Non-synm	C	G	Transversion	H180D
9	T556A	Non-synm	T	A	Transversion	C186S
10	A578G	Synm	A	G	Transition	E193G
11	T584C	Non-synm	T	C	Transition	L195P
12	C602T	Non-synm	C	T	Transition	S201F
13	G626C	Non-synm	G*	C	Transversion	S209T**
14	C627G	Non-synm	C*	G	Transversion	-do-
15	G628A	Non-synm	G	A	Transition	G210R
16	T635G	Non-synm	T	G	Transversion	M212R
17	T743A	Non-synm	T	A	Transversion	V248E
18	C778G	Non-synm	C	G	Transversion	L260V
19	C783G	Synm	C	G	Transversion	
20	G790C	Non-synm	G	C	Transversion	A264L
21	C791T	Non-synm	C	T	Transition	A264L
22	G795C	Synm	G	C	Transversion	
23	T798G	Synm	T	G	Transversion	
24	G799T	Non-synm	G	T	Transversion	A267C
25	C800G	Non-synm	C	G	Transversion	A267C
26	C1058T	Non-synm	C	T	Transition	S353F
27	C1087T	Synm	C	T	Transition	

*This nucleotide is present in cattle sequences gene bank Acc no. EU 148609, EU148610, EU148611, whereas cattle sequences NM174008, AF141313 contain the nucleotide sequence as in crossbred cattle. This SNP is already reported.

**Amino acid Serine is present in cattle sequences protein id Acc no. AAD32215, ABV68571, ABV68570, ABV68569, NP776433, ADB92696 (CB cattle).
